# Recent Advances in Thermoplastic Microfluidic Bonding

**DOI:** 10.3390/mi13030486

**Published:** 2022-03-20

**Authors:** Kiran Giri, Chia-Wen Tsao

**Affiliations:** Department of Mechanical Engineering, National Central University, Taoyuan City 320, Taiwan; 110323603@cc.ncu.edu.tw

**Keywords:** microfluidic chip fabrication, microfluidic bonding, thermoplastic bonding, polymer microfabrication

## Abstract

Microfluidics is a multidisciplinary technology with applications in various fields, such as biomedical, energy, chemicals and environment. Thermoplastic is one of the most prominent materials for polymer microfluidics. Properties such as good mechanical rigidity, organic solvent resistivity, acid/base resistivity, and low water absorbance make thermoplastics suitable for various microfluidic applications. However, bonding of thermoplastics has always been challenging because of a wide range of bonding methods and requirements. This review paper summarizes the current bonding processes being practiced for the fabrication of thermoplastic microfluidic devices, and provides a comparison between the different bonding strategies to assist researchers in finding appropriate bonding methods for microfluidic device assembly.

## 1. Introduction

**Figure 1:**
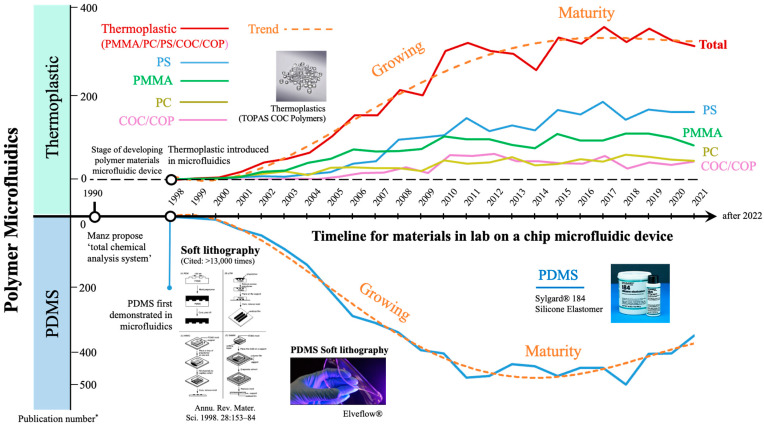
Development trends of PDMS and thermoplastic in microfluidics. * The publication numbers are analyzed by the Web of Science website and the data were collected until December 2021.

Microsystem Technology (MST) is a multidisciplinary technology that integrates electronics, mechanical, optical, or chemical devices in a miniaturized system. Microfluidics, which was derived from MST and involves fluidic components, has developed rapidly since its introduction by Manz et al. in 1990 [1]. Over 30 years of development until this date, microfluidics, or its relative terms, such as micro total analysis system (μTAS), or lab-on-a-chip device (LOC) have been widely applied in various fields [2,3]. In the early stage, when microfluidics was developed from semiconductor technology, silicon and glass were the major materials for device fabrication. With microfluidic manufacturing progression, polymer materials have gradually replaced silicon materials, primarily because of their simple, low-cost, and disposable advantages. In polymer microfluidics, polydimethylsiloxane (PDMS) and thermoplastic are the two major materials, and both materials present their own unique qualities in microfluidic applications. As displayed in Figure 1, since the first introduction of PDMS and thermoplastic in microfluidics around 1998, both materials have grown rapidly, reaching their maturity stage around 2014. Currently, both PDMS and thermoplastic play an important role in microfluidic device commercialization [4].

PDMS is an elastomer material that can be deformed under force or pressure application. The invention of the PDMS microvalve and PDMS pump has also allowed complex fluidic manipulation and very large-scale integration [5,6]. Due to its high gas permittivity with optical transmissivity, PDMS is an attractive material for cell-based microfluidics or organ-on-chip applications [7,8,9]. In addition, the fabrication of PDMS devices by the soft lithography process is also straightforward and reliable, which enables researchers to quickly and easily implement PDMS microdevices in real applications [10]. Despite the aforementioned advantages, PDMS still possesses several limitations for microfluidic applications. Problems such as solvent swelling, hydrophobic molecule absorption, lack of mechanical rigidity or low fabrication throughput are the fundamental challenges associated with the PDMS material [8,11,12].

Thermoplastics are among the most widely used engineering polymers that have been applied in a wide range of applications. Compared to PDMS, thermoplastics possess good mechanical rigidity, organic solvent resistivity, acid/base resistivity, and low water absorbance. In addition, thermoplastics are synthetic polymers that have various surface properties for microfluidic applications. Thermoplastics, such as poly (methyl methacrylate) (PMMA), polycarbonate (PC), polystyrene (PS), or cyclic olefin polymer/copolymer (COC/COP), have been widely used in polymer microfluidics (Figure 1). Table 1 summarizes the aforementioned thermoplastic properties for use in microfluidic applications. These advantages make thermoplastic ideal for many bioanalytical applications.

In thermoplastic microfluidic chip fabrication, various methods for creating microchannels or microstructures on the thermoplastic substrate have been demonstrated. Rapid prototyping methods, such as CNC milling [16], laser ablation [17,18], and recent 3D printing technologies [19,20], are available for researchers to proof-of-concept at small production volumes. Replication methods, such as hot embossing/imprinting [21], roller imprinting [22,23], injection molding [24,25], and thermoforming [26] methods, are practiced to mass-produce the thermoplastic chips. These versatile front-end fabrication capabilities and high production throughput make thermoplastics a favorable material choice alternative to PDMS. 

Similarly to microchannel fabrication, post-end thermoplastic bonding also exhibits fabrication diversity. Becker et al. [4,27] and Tsao et al. [12,28] have organized and reviewed various thermoplastic bonding methods for polymer microfluidics. The post-end “world-to-chip” interfacing is also summarized by Temiz et al. [29]. However, unlike PDMS bonding, thermoplastic bonding is relatively complex, which requires the selection of the most appropriate bonding process based on the application requirements. Up to the current date, thermoplastic bonding is still an intriguing research topic because of the need to have a reliable, simple, and robust bonding method. In this review paper, we aim to provide reviews of current thermoplastic bonding technologies and targets to assist researchers in selecting an appropriate bonding method for their thermoplastic microfluidic chip fabrication design. 

### Thermoplastic Bonding Strategies in Microfluidics

The thermoplastic bonding methods in microfluidics can be categorized into direct and indirect approaches [28]. Direct bonding is defined as the process in which the thermoplastic pairs are “directly” bonded without additional material or reagent layer in the bonding interface. Bonding methods such as thermal fusion bonding, solvent bonding, physical surface modification, and ultrasonic/laser welding are categorized as direct bonding methods. Indirect bonding, on the other hand, is defined as the bonding that involves using additional material, such as epoxy, adhesive tape, or chemical reagents to assist the bonding. Thermoplastic bonding methods, such as adhesive bonding, chemical surface modification, and microwave bonding that uses adhesives, chemical reagent, or metal layers, are categorized as indirect bonding methods. 

Several important factors should be considered for determining the thermoplastic bonding process. Bonding strength is one of the critical factors that should be considered in the bonding process. Bonding strength should be strong enough to hold the thermoplastic chip without leakage during microfluidic chip flow injection or operation procedures. For general microfluidic handling and transportation conditions, low-to-medium bonding strength is sufficient for holding the chip. While operating at a high flow rate, or operating in the high hydraulic resistance microchannel, such as with porous microstructures or high-density microchannels, medium to high bond-strength bonding methods should be considered. In many microfluidic applications, such as cell culturing or DNA/protein separation/identification, observation of cell or fluorescence images under a microscope is required. Therefore, optical transmissivity should be considered while selecting an appropriate bonding method. In addition, since microfluidic devices are currently in the maturity stage, they translate the technology from the research lab to commercial productions [4]. Low-volume (<250 pieces per month) thermoplastic bonding production rates may be appropriate for academic or research labs developing prototypes or proof-of-concepts, while for commercialized microfluidic devices, medium-volume (250~1000 pieces per month) or high-volume (>1000 pieces per month) mass production thermoplastic bonding strategies should be selected. Therefore, in addition to the mechanical and optical properties of the bonding interface, fabrication throughput is particularly important while considering the commercialization of the device. Each thermoplastic bonding detail is reviewed and discussed in the following sections. 

## 2. Direct Bonding

### 2.1. Thermal Fusion Bonding

**Figure 2:**
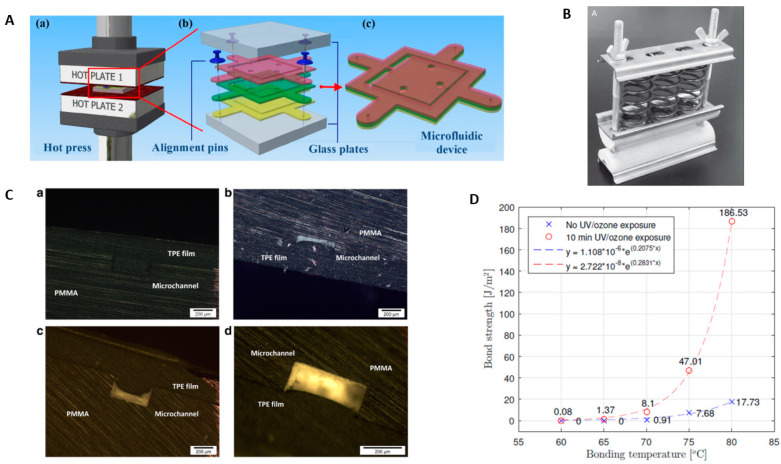
(**A**) Schematic of the thermal fusion bonding process. Reprinted with permission from ref. [30]. Copyright 2015 IOP Publishing Ltd. (**B**) Image of a spring-driven press device for hot embossing and thermal bonding of PMMA microfluidic chips. Reprinted with permission from ref. [31]. Copyright 2010 John Wiley and Sons. (**C**) Optical micrographs of PMMA microchannel in PMMA to Thermoplastic Elastomer (TPE) joint after bonding at (**a**) Under 5.23 MPa. (**b**) Under 2.61 MPa. (**c**) Under 0.78 MPa. (**d**) Under 0.52 MPa. pressure condition. Reprinted with permission from ref. [33]. Copyright 2016 Elsevier. (**D**) Bond strength results of the wedge test for different times of UV/ozone exposure [34].

Thermal fusion bonding (also called thermal bonding or thermopress bonding) is a process of sealing microfluidic devices by heating the thermoplastic substrates near or above their glass transition temperature (T_g_) and simultaneously applying pressure. The thermoplastic becomes rubbery when heated near or above its T_g_ and can deform upon application of pressure. After heating and applying pressure, the polymer chains “fuse” together through inter-diffusion at the bonding interface. Thermal bonding is a simple process, and has the ability to develop homogenous microchannel surfaces after bonding, due to the absence of intermediate bonding materials. Because of the simple bonding mechanism and various advantages, it is the widely used method for sealing thermoplastic microfluidic chips. During the thermal bonding, the thermoplastic pairs are aligned between glass pairs, and bonded by applying pressure and heat with the help of hot embossing or hot press machine, as illustrated in Figure 2A [30]. In addition to conventional hot press machines or bonders, various low-cost bonding facilities have also been utilized to achieve thermal fusion bonding. A low-cost flexible spring-driven embossing/bonding device (Figure 2B) has been successfully designed and fabricated for the development of PMMA microfluidic chips. The pressure for embossing and bonding can be adjusted by fastening and loosening the screw nuts [31]. Wang et al. also developed a positive temperature coefficient ceramic heater accompanied by spring-driven press to bond PMMA microfluidic chips [32]. 

Since thermal fusion bonding is achieved by heating the thermoplastic substrates above T_g_, a rapid decrease in elasticity modulus at high temperature causes the deformation of the microchannels. Optimal bonding parameters (temperature, pressure, time, etc.) are required for appropriate bonding of thermoplastic microfluidic devices. The parameters selected should be able to bond with the required bond strength without the distortion of microchannels. As the optical micrographs displayed in Figure 2C, the higher pressure results in clogging of the microchannels compared to the low pressure conditions. The parameters appropriate for bonding thermoplastic elastomer (TPE) and PMMA is found to be temperature 80 °C, time 30 min and pressure 0.42 MPa [33]. Different methodologies have been practiced to reduce high-temperature and microchannel distortion issues during thermal bonding. Wang et al. established a numerical quasi-creep model to predict the appropriate temperature for bonding of thermoplastics for lower deformation [35]. A new bonding method was proposed, which used two different layers of the same thermoplastic material PS with different T_g_ and obtained the deformation ratio of 1.1%, which is significantly smaller than the traditional thermal bonding process [36]. The thermal bonding of PMMA substrates was achieved by heating in a vacuum-heating oven for 60 min at 112 °C under the vacuum pressure of 10 mbar, which led to a tight assembly at the interface between the two PMMA sheets, with well-preserved dimensions in the channels [37]. Mahmoodi et al. suggested the use of GATB (Gas-Assisted Thermal Bonding), in which pressurized gas is used to supply the uniform force to bond the two substrates. This process resulted in less-distorted microchannels with good channel-geometry integrity, because of isostatic pressure distribution applied to bonding surfaces [38]. A pressure-assisted boiling point control system was developed to apply precise temperatures and pressures during thermoplastic fusion bonding of PMMA substrates without deformation of microchannels [39]. Thermal bonding can be used for sealing microchannels using ordinary polyimide (T_g_ = 345.7 °C) with no additives and special functions, at a temperature of 380 °C when the contact time is 5 min, and 390 °C when the contact time is 3 min [40].

The high temperature during thermal bonding causes the change in the mechanical properties of the material, which results in the deformation of the microchannels under force application. In order to obtain high-quality microchannels, the temperature for bonding should be reduced. Plasma, ultraviolet (UV) radiation, or chemical treatment can help in reducing the bonding temperature of thermoplastics, as well as improving the bonding strength in thermoplastic bonding. For example, as illustrated in Figure 2D, the high bonding strength is obtained at a temperature below the T_g_ after UV exposure for 10 min [34]. Zhu et al. found that thermal bonding after surface modification can achieve successful bonding at lower bonding temperature and pressure, which can protect the microstructures during the bonding process [41]. The two PMMA substrates exposed briefly to an oxygen plasma were assembled by thermal bonding, at a temperature close to T_g_ (i.e., 108 °C) and under a pressure of 100 bar [42]. UV treatment before thermal bonding has been shown to achieve an increase in bonding strength and reduced bonding temperature [34,43,44]. O_2_ plasma and ethanol treatment prior to thermal bonding has been explored as an alternative to conventional O_2_-plasma-assisted thermal bonding to reduce the distortion of nanochannels [45]. Thermal bonding assisted by O_2_ plasma and ethanol reduces deformation, due to the bonding occurring at lower temperature and pressure and over a shorter time. To bond COC substrates (T_g_ = 178 °C) with PMMA (T_g_ = 105 °C), the T_g_ of COC was reduced to 75 °C via UV/ozone activation or O_2_ plasma treatment, and bonded at 5 °C below the T_g_ of COC substrates [46]. Plasma-treatment-assisted thermal bonding has been practiced to increase the strength of bonding in thermoplastic microfluidic devices, and also to reduce the temperature for bonding [33,45,47,48]. Oxygen plasma treatment of surfaces makes them hydrophilic, enhancing the bonding strength of microfluidic devices [33,48]. Surface-confined carboxylic acids were generated from oxygen plasma treatment, which helped in thermal bonding at a low temperature [48]. 

In addition to process parameter control and combination with surface modification techniques, other thermal bonding methodologies are also practiced to improve bonding quality and solve microchannel clogging issues. Du et al. used water pretreatment of PMMA substrates prior to thermal bonding to increase bonding rate by 34% compared to conventional thermal bonding [49]. Gong et al. developed an interference-fit-assisted thermal bonding, which enables bonding at low pressure and reduces bond defects. This process allows bonding of thermoplastics within a wide range of bonding pressure levels with easier process optimization [50]. Solvent-assisted bonding has shown improvement in the bond strength of the microfluidic device due to the extra roughness of the surface, which prevents air traps and increases the contact area. The treatment of surfaces using boiling isopropanol also helped to smooth the coarse microchannels produced by CO_2_ lasers, without altering the properties of bonding surfaces [51]. Another method using vacuum bagging technique with thermal bonding has been explored for producing thermoplastic microfluidic devices. This method provides high-strength bonding at a lower temperature and makes it possible to assemble multiple layers with a single step [52]. 

The thermal bonding methods, parameters, tools used, and bonding results are summarized in Table 2. The bonding temperature, pressure, and time are the important parameters in thermal bonding. Thermal bonding mostly occurs at temperatures near or above T_g_. However, the surface treatment method enables thermal bonding below T_g_. For PMMA, the bonding temperature ranges from 90 °C to 170 °C. Pressure of up to 6.9 MPa is required to obtain thermopress bonding of the thermoplastic substrates. The processing time for bonding ranges from 1 min to 1 h. A hot embossing machine [45], a hot press machine [44], or a low-cost spring-driven press [31] can be used for providing heat and pressure for the thermal bonding process. UV treatment, plasma treatment, water treatment, etc. are used to improve the bonding quality of microfluidic devices. A bonding rate of 95.3% [49] and bonding strength of up to 808.0 ± 80 kPa [51] can be obtained by the thermal bonding process for PMMA substrates.

### 2.2. Solvent Bonding

**Figure 3:**
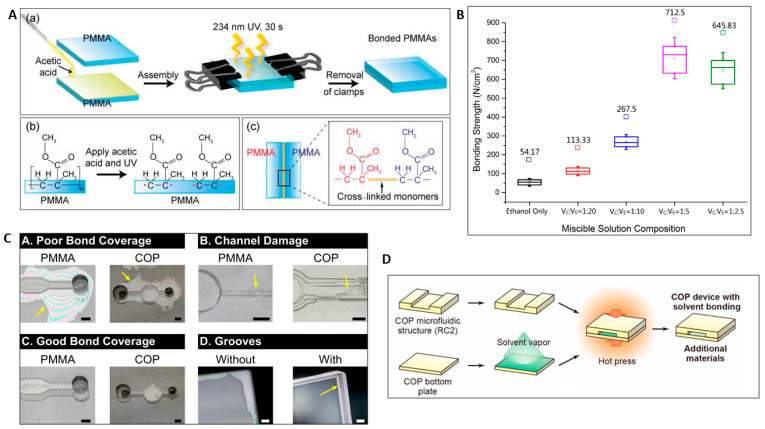
(**A**) (**a**) Schematic diagram of UV-assisted liquid solvent bonding process. (**b**,**c**) Chemical reaction on PMMA substrates after solvent and UV treatment. Reprinted with permission from ref. [53]. Copyright 2020 American Chemical Society. (**B**) Bonding strength at different chemical composition of chloroform and ethanol [54]. (**C**) Different defects in solvent bonding and solutions. Reprinted with permission from ref. [55]. Copyright 2017 JoVE (**D**) Schematic of the cyclo-olefin polymer (COP)-based microfluidic device fabrication process by vapor solvent bonding [56].

Solvent bonding is a widely practiced method for permanent bonding of thermoplastics for microfluidic application. It is a commonly used technique because of its advantages, such as low cost, good optical clarity, fast bonding at low temperature, and particularly for its high bond-strength performance. This method involves applying the solvent as a liquid or vapor phase on the bonding surface, which dissolves the surface layer of the polymer substrate and the solvated layer then acts as “adhesive” for bonding. The solvent molecules must overcome the Van der Waals forces between the polymer molecules on each bonding surface for solvent bonding to occur, which results in higher degrees of freedom for the polymer chains. When the two solvent-softened surfaces are brought into contact, the polymer chains from the two surfaces bond with each other. After the solvent starts evaporating, the entangled chains become more and more constrained and stop the polymer motion entirely after complete evaporation of the solvent. A liquid solvent bonding process and chemical reaction on the surface of PMMA substrates during solvent bonding is shown in Figure 3A [53]. Liquid solvent is added between the two thermoplastic substrates layers to be bonded and the layers are brought together. Low pressure and temperature (below T_g_ of substrates) are applied to the device for the required duration, and the bonding is complete. Solvent bonding of PMMA substrates was achieved using acetic acid as a solvent at room temperature, with clamp-assisted low pressure with only 30 s of the UV irradiation [53]. 

Microchannel clogging and distortion are the fundamental challenges in solvent bonding. The solvent assists in the bonding of thermoplastic microfluidic devices, but excessive solvent causes solvated polymer reflow, resulting in clogging or collapsing of the microchannel. Therefore, the solvent composition and bonding parameters must be closely controlled to prevent deformation or clogging of the microchannels. Generally, solvent uptake should be kept low to reduce channel distortion of the microfluidic device during the bonding process. This is controlled by the solvent exposure time; however, it needs to be balanced by the required bonding strength and bonding pressure [57]. Ng et al. proposed controlling the process parameter by using a thermally activated solvent bonding process. The bonding is based on the temperature-dependent solubility of polymer in a liquid that is not a solvent at room temperature, but which after thermal activation becomes transformed into a solvent of the polymer, creating a chain interdiffusion at the bonding interface [58]. However, in most cases the effective solubility of solvents for bonding different thermoplastics is obtained by mixing different solvents. The effect of miscible solution composition (chloroform and ethanol), and the tensile test results are shown in Figure 3B. The increase in volume of the chloroform results in improvement of the bonding strength up to a maximum strength of 712.5 N/cm^2^ when the volume ratio is V_C_:V_E_ = 1:5 (about 13 times larger than when there is only ethanol in the solution) [54]. Yin and Wang proposed a novel bonding method based on acetone-and-ethanol- (v:v, 8:2) treatment on a PMMA surface to adjust the Young’s modulus in its surface layer. PMMA substrate treated with the solution for 30 s showed an increase in Young’s modulus with minimum distortion in nano-trenches [59]. Faghih and Sharp conducted a study to test the bonding of PMMA using different solvent mixtures and solvent phases (i.e., liquid vs. vapor), curing times and temperatures, and the effect of corona surface treatment on bonding strength. A solvent mixture of 20% dichloromethane and 80% isopropanol showed the best bonding quality, while vaporized dichloromethane had the best optical transparency. The corona surface treatment showed a 25% increase in bonding strength and 2% improvement in optical clarity using 20% dichloromethane and 80% isopropanol solution at room temperature and a curing time of 15 min [60]. Ultra-fast bonding of PC substrates was obtained using acetone and n-pentane in the solution, in which n-pentane acted as a sacrificial solvent, and acetone acted as the solvating solvent [61]. Keller et al. found that 35 vol% cyclohexane in acetone gives the best result for clarity while bonding COC substrates [62].

In addition to finding an optimized solvent composition for bonding, other methods are practiced to reduce clogging and distortion of the microchannel. Gan et al. proposed a solvent bonding process that used a phase-changing agar hydrogel as a sacrificial layer for the fabrication of PMMA microfluidic devices. The melted agar hydrogel was placed in channels and the reservoir ports before the bonding process, which sets at room temperature, and form a solid layer and prevents solvent and softened PMMA surface from filling the channels. After bonding, the agar hydrogel was melted and removed from channels and the reservoir [63]. Rahbar et al. developed a novel low-cost bonding method combining microwave bonding and thermally assisted solvent bonding. The solvent was applied to two PMMA bonding surfaces and clamped using binder clips and placed in microwave for 90 s. Ethanol was found to be most effective for bonding of PMMA substrates, and channel clogging and distortion was reduced [64]. Microwave assisted bonding using acetic acid as solvent was also used to achieve uniform bonding and high bond-strength without using any external pressure while bonding PMMA substrates [65]. Ng et al. developed a bonding technique to prevent excessive channel clogging and distortion, in which the PMMA substrates were bonded by isopropanol combined with the pre-processing step of pressure and thermal annealing of the PMMA substrates, and a post-processing step of solvent removal by subjecting the chip to a vacuum environment. These pre and post-processing steps produced a chip with better optical clarity, and had strong bonding strength and minimal distortion and clogging of the microchannels [57]. 

Another major challenge in liquid solvent bonding is the occurrence of rapid evaporation near the free edges of the chips during the heated bonding step, due to solvent volatility. This often causes poor bonding coverage, leakage in devices and reduced bonding strength due to regional unbonded areas near these edges [55]. The problem of poor bonding of the substrates near the edges was mitigated by making solvent retention grooves near the edges of microfluidic devices. Figure 3C [55] illustrates the challenges in solvent bonding and the solutions. Poor bonding near the edges occurs due to rapid evaporation at the free edges of the microfluidic device during heated bonding, whereas overly aggressive solvent bonding causes clogging in microchannels. Therefore, an optimized solvent-thermoplastic system is required to obtain good bond quality and reduce the deformation in microchannels, and solvent retention grooves parallel to the device edges can help to improve bond strength. Wan et al. developed a novel bonding method that uses the retention grooves at the bonding surface to achieve a uniform liquid-phase solvent bonding of thermoplastic microfluidic devices. This method alleviates evaporation effects and thus allows the use of liquid-phase solvent bonding to obtain high-quality bonds for various thermoplastics and solvents [66]. Bamshad et al. demonstrated that a 70% solution of isopropyl alcohol solvent with one-step cooling produced the best bonding result with a bonding strength of 28.5 MPa, while bonding PMMA substrates using thermal solvent-assisted bonding method [67]. Lynh and Chuan fused the two materials with similar solubility parameters. PMMA and PLA, using ethanol as a solvent, were allowed to cross-link through UV exposure, followed by annealing to remove residual stress [68]. 

In solvent application, immersion and dropping of solvent on the microfluidic chip are unable to produce uniformity in the distribution of the solvent. Therefore, Chen et al. suggested the spin-coating method for uniformity in the distribution of ethanol solution [69,70]. Solvent bonding with spin coating was used in the development of micromixers. Pure isopropyl alcohol at 70 °C was applied on the bottom surface of PMMA for 10 s using a spin coater at 2000 rpm [71]. Duong et al. proposed a spray coating method for uniform distribution of ethanol solution to bond PMMA and ABS substrates. The bonded surfaces were exposed to UV radiation for proper bonding, and post-annealing was conducted after UV exposure to reduce residual stress and increase bond strength [72]. Nemati et al. developed a new method that focused on interfacial polymer-solvent bonding. An amplified bonding strength was attained due to precise polymer dissolution, which was strictly constrained at the polymer-solvent interface [73]. 

Alternatively to the liquid solvent bonding, vapor solvent evaporation is extensively used in solvent bonding for uniform solvent deposition, resulting in a clogging- and distortion-free microchannel. In vaporized solvent bonding, solvent is applied on polymer substrates, which softens the surface of the polymer. Then, the excess of solvent remaining on the surface is allowed to evaporate until the surface re-solidifies. Then, two polymer pieces are pressed together and bonded [74]. The schematic diagram of the solvent vapor bonding for a COP-based microfluidics device is shown in Figure 3D [56]. Wouters et al. used cyclohexane solvent-vapor-assisted bonding to reduce the clogging of microchannels observed while bonding COC substrates with the cyclohexane solvent [75]. Rodriguez et al. used chloroform vapor to form a weak bond between two PMMA layers. The channel network layer was bonded using methylene chloride, which was exposed to the bottom layers for 15 s for bonding [76]. Akhil et al. proposed bonding of PMMA substrates with chloroform solvent using vaporized solvent bonding. An exposure time of 60 s yielded the highest quality bond. Unmodified PMMA substrates yielded the best results, due to higher molecular weight than UV-radiation-exposed PMMA surfaces [74]. Sun et al. developed a thermoplastic bonding process that used exposure of the PMMA substrate to chloroform vapor, followed by a low-temperature vacuum thermal bonding below the PMMA’s T_g_ [77]. 

The solvent bonding methods, parameters, tools used, and bonding results are summarized in Table 3. Different solvents are used based on the solubility properties of thermoplastics and the required characteristics for microfluidic devices. For instance, PMMA substrates are bonded with different solvents, such as ethanol [67,69], chloroform [77], and isopropyl alcohol [71], as well as miscible solution of chloroform-ethanol [54], dichloromethane-isopropanol [60], etc. Bonding can be obtained at low temperature and ordinary pressure in solvent bonding. However, solvent exposure time ranges from 10 s to 20 min. Longer exposure time increases the bonding strength of the microfluidic device. For application of the solvent to the substrates, different methods are practiced. The solvent can be spread uniformly on thermoplastics by pipette [57], or by other methods, such as spray coating [72], soak method [54], and spin coating [71]. Plasma treatment [77] and UV irradiation [72,74] methods are used to improve the bonding quality. A very high bond strength of up to a maximum of 38 MPa at low temperature and under 1 atmospheric pressure can be obtained for PMMA substrates while bonding through the solvent bonding method [77].

### 2.3. Physical Surface Modification

**Figure 4:**
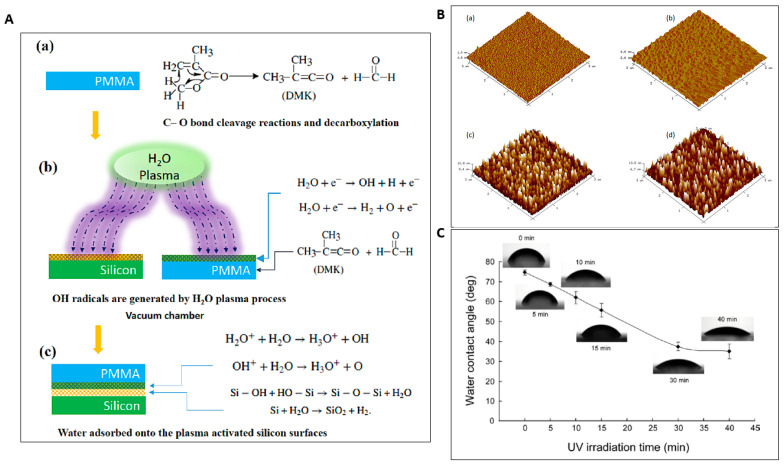
(**A**) Chemical structure of PMMA; chemical reaction during and after H_2_O plasma treatment while bonding between PMMA/silicon substrates [79]. (**B**) Surface roughness of PMMA and silicon before and after H_2_O plasma treatment [79]. (**C**) Water contact angle measured after UV irradiation on the PMMA substrate over time. Reprinted with permission from ref. [84]. Copyright 2015 IOP Publishing Ltd.

The surface treatment of thermoplastic is used for bonding thermoplastic microfluidic devices, as well as for enhancing the bonding strength between two substrates. This is attained by increasing the surface energy of the thermoplastic substrates, which results in an enhancement of the hydrophilicity of the surface. Surface treatment and modification for thermoplastic microfluidics are normally achieved by either physical or chemical approach. The physical modification methods use UV or plasma treatment to render the thermoplastic surfaces. There is no additional material or reagent at the bonding interface. Therefore, physical surface modification bonding is categorized as a direct bonding approach. The physical surface treatments mainly produce polar functional groups that can assist in the formation of strong covalent or hydrogen bonds. They also cause polymer chain scission, which decreases the molecular weight, resulting in a decrease in T_g_, thereby helping the adhesion of thermoplastic microfluidic devices bonding at low temperature. After the polymer is treated with plasma, polar components, such as carboxyl (–COOH), carbonyl (–C=O), amidogen (–NH_2_), and hydroxyl (–OH) are generated on the surface of the polymer. A study of plasma treatment on PMMA surfaces showed that surface modification occurs in two steps: first, ablation of the outer surface, and second, increase in oxidation of the PMMA surface by the formation of an O-C-O group [78]. The surface treatment breaks the original chemical bonds on the polymer surfaces and generates polar components at the surface, which help in the bonding of the surface. The chemical structure prior to and after H_2_O plasma treatment and the chemical reaction during the bonding are shown in Figure 4A [79]. The study of UV radiation in the presence of oxygen gas or plasma revealed that the polar functional groups, such as -OH and -COOH, are generated on the surface and assist in forming bonds on COC substrates. Degradation on the surfaces was also observed, and helped in the formation of direct bonds between the surfaces [80]. The amount of hydroxyl can decrease rapidly after plasma treatment, since hydroxyl can react with oxygen in the atmosphere. Therefore, Qu et al. proposed the use of water after plasma treatment, as it acts as an inhibitor of hydroxyl, prevents reactions between hydroxyl and atmospheric oxygen, and increases bonding rate and strength [81]. Surface modification results in an increase in surface hydrophilicity [82], which was found to have a significant effect on the strength of the bonds formed by thermal and solvent bonding processes [83].

Physical surface treatment bonding is found to provide the required bonding strength for microfluidic devices. Several physical modification methods are practiced for bonding thermoplastic substrates with high-bonding strength. Song et al. used oxygen plasma treatment followed by annealing for reversible adhesion of PDMS with PS for microfluidic cell culture applications [85]. H_2_O plasma treatment followed by Rapid Thermal Annealing (RTA) was used to bond PMMA based microfluidic devices [86]. Terai et al. used water-vapor-assisted plasma treatment in the adhesion of COP and glass substrates, which also helped to maintain stable superhydrophilicity i.e., water contact angle <1° [87]. Oxygen-plasma treatment results in a reduction in the water contact angle of polyethylene terephthalate (PET) substrates from 82.1° to 37.6°, and bonding was obtained at low temperature and under low pressure [88]. Tsao et al. achieved low-temperature bonding of thermoplastic microfluidic substrates with control of hydrophilicity by modification of PMMA and COC surfaces with UV/ozone [89]. 

Plasma treatment is one of the most prominent methods in physical surface modification bonding, which increases the bond strength by increasing the hydrophilicity and the surface roughness of bonding substrates. As shown in Figure 4B [79], the surface roughness (R_max_) of the untreated PMMA and silicon was around 1.68 nm and 2.23 nm, respectively, which increased to 14.9 nm for PMMA after a 30 s plasma treatment time, and 30.2 nm for silicon after a 90 s plasma treatment time. Therefore, strong adhesion between heat-assisted plasma-treated fluoropolymers (PTFE, PFA) and plasma-jet-treated PDMS was obtained. The plasma-modified surfaces formed hydrogen and covalent bonds (C–O–Si and/or C(=O)–O–Si) between hydroxyl (C–OH) and carboxyl (C(=O)–OH) groups of the treated PTFE [47]. Roy et al. studied the effect of plasma treatment on thermal bonding in COC-based microfluidic devices. Argon and oxygenated argon plasma showed improvement in hydrophilicity (from 86° to 17° and 14°, respectively) and surface roughness of COC substrates. However, a significant decrease in wettability was found when plasma treated surfaces were exposed to air for long time [90]. Plasma treatment increased the hydrophilicity (water contact angle dropping from 60° to less than 5°) and surface roughness of the PMMA surface, and improved the electrokinetic performance of the microfluidic device, resulting in an electroosmotic flow(EOF) value of 2.83 ∗ 10^−4^ cm^2^/Vs [82]. Plasma surface modification was also used to first remove the fracture of microelectrodes of a PMMA microfluidic chip and decrease bonding temperature from 100 °C to 85 °C [91]. Use of plasma surface modification along with TEOS treatment can remove the need for strong solvent and high temperature for the bonding of microfluidic devices [92]. Plasma treatment of 4 min on a COC surface decreased the contact angle from 88° to 7°, which enhanced bonding strength [93]. Oxygen plasma treatment was also used to assist the bonding of PMMA and PDMS layers at room temperature using biocompatible adhesive tape [94]. While bonding TPE and PMMA substrates, O_2_ plasma treatment is found to be more effective in improving the bonding strength when applied on the surface of a TPE film than on a PMMA surface [33]. 

UV/ozone surface treatment is another commonly used physical surface modification method. Similar to O_2_ plasma treatment, the UV/ozone treatment is used for increasing surface energy (decrease in water contact angle) of thermoplastic substrates. Figure 4C illustrates the decrease in water contact angle with the increase in UV irradiation time [84]. UV/ozone treatment was used to modify thermoplastic substrates by exposing them to UV light with wavelengths of 184.9 nm and 253.7 in an air-filled chamber. UV light at 184.9 nm decomposes oxygen molecules and synthesizes ozone, whereas UV light at 253.7 nm decomposes ozone molecules, rapidly oxidizing hydrocarbons and producing high-energy oxygen radicals on the thermoplastic surface [89]. UV/ozone surface treatment helps to reduce the bonding temperature for thermopress bonding [34]. UV light from an excimer lamp with 172 nm wavelength and 10 mW/cm^2^ intensity was used to break polymer chains on thermoplastic while bonding TPE with PMMA surfaces [44]. Fan et al. found that deep-UV surface modification of PMMA and PS contact surfaces can enhance the bonding strength in thermopress bonding at the same temperature and pressure, but also causes a yellowing effect, which reduces the optical clarity of thermoplastic surfaces [95].

### 2.4. Ultrasonic and Laser Welding

**Figure 5:**
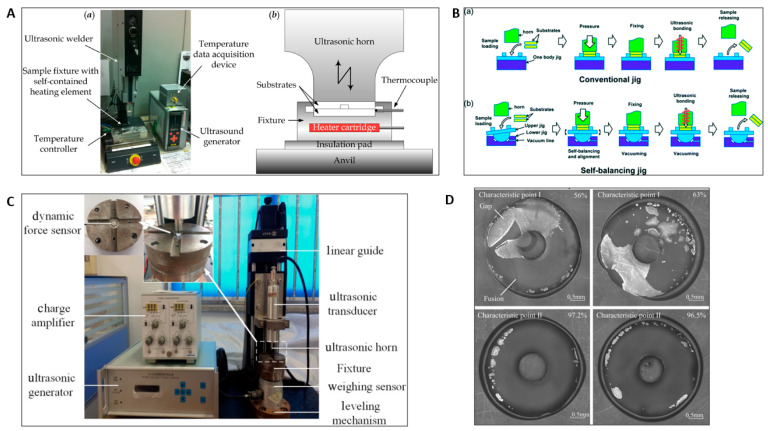
(**A**) (**a**) Ultrasonic bonding complete setup. (**b**) Schematic diagram of ultrasonic bonding apparatus. Reprinted with permission from ref. [97]. Copyright 2014 IOP Publishing Ltd. (**B**) Self-balancing jig apparatus. Reprinted with permission from ref. [100]. Copyright 2015 Royal Society of Chemistry. (**C**) Ultrasonic bonding test bench. Reprinted with permission from ref. [101]. Copyright 2019 John Wiley and Sons. (**D**) Interfacial fusion at the two characteristic points Reprinted with permission from ref. [101]. Copyright 2019 John Wiley and Sons.

Both ultrasonic and laser welding are weld-bonding processes that do not require foreign substances, such as adhesives or solvents. They can either fully bond the thermoplastic substrates or weld locally. Ultrasonic bonding involves the bonding of plastic by local melting caused by the propagation of ultrasonic sound at 20~40 kHz or higher between a sonotrode (weld horn) and an anvil [96]. The vibrations produced by the sonotrode are concentrated in the bonding area through the means of energy directors. The intermolecular and boundary friction generate the heat required for the fusion of the thermoplastics [96]. The ultrasonic welding setup and schematic of the different components (i.e., ultrasonic horn, anvil, and fixture) of an ultrasonic bonding apparatus are shown in Figure 5A [97]. The study of the heating process of ultrasonic welding for thermoplastics revealed that the welding process is started by interfacial friction, and is continued by viscoelastic heating after the temperature reaches T_g_ [98]. Ultrasonic bonding is a rapid and low-temperature bonding process that yields joints with high bond strength. It is also effective when used for bonding very thin layers of various materials, due to the rapid bonding process. Ultrasonic bonding has short cycle times and is comparatively economical, and therefore is preferred for mass production of microfluidic chips [99]. 

Ultrasonic bonding methods have been practiced for different thermoplastic bonding purposes at low temperature. Li et al. presented a bonding method for batch production of PMMA microfluidic devices using an ultrasonic field with high bonding strength of up to 30 mJ cm^−2^ at only 60 °C, and low channel distortion [102]. The preheated COC substrates were bonded at a temperature 20 °C lower than its T_g,_ with negligible microchannel distortion, by application of longitudinal ultrasonic actuation, and the bond strength obtained was comparable to the bond strength obtained from thermopress bonding at 5 °C above the T_g_ [97]. Zhang et al. demonstrated a thermal-assisted ultrasonic bonding method in which bonding was obtained at 20–30 °C below the T_g_ of PMMA substrates using low amplitude ultrasonic vibration. The rapid bonding with high-strength bond (0.95 MPa) and low dimension loss (0.3–0.8%) was obtained due to bonding at low temperature and low pressure [103]. Kistrup et al. demonstrated the fabrication of microfluidic chips with a bonding time of 1 min per chip by combining injection molding with ultrasonic bonding [104]. Qui et al. proposed an ultrasonic welding method for multilayer bonding of PMMA and ductile PC. The interposed sheet (IPS) prepared from three layers of functional gradient materials was used for welding [105]. Zhang et al. demonstrated a novel ultrasonic welding process assisted by isopropanol to bond PMMA microfluidic chips [106]. 

Ultrasonic bonding has some limitations, such as difficulty in adjusting microchannel heights due to shrinkage of polymers, and partial or excessive fusion due to uneven bonding energy distribution. Different solutions have been suggested to mitigate these limitations. Li et al. proposed the designing of the energy director’s structure by studying relationship between microchannel heights and bonding parameters for controlling the height of the microchannels and reducing the clogging of microchannels [107]. Ng et al. showed that the use of inserts can prevent blockage due to the flow of melt material while bonding connectors to the microfluidic device using ultrasonic welding [108]. Lee et al. developed a hemisphere-shaped self-balancing jig, which assists in precisely adjusting and sealing the heterogeneous microstructure during ultrasonic bonding [100]. Different bonding processes using conventional and self-balancing jigs are demonstrated in Figure 5B. The self-balancing jig properly aligns the microfluidic device and horn to minimize the bonding failure [107]. For ultrasonic bonding, the energy directors are complicated and make it difficult to control the molten polymer reflows. Therefore, some alternatives are proposed as substitutes for the energy director for ultrasonic bonding. Liang et al. suggested an alternative in which a small bulge is formed at the edge of the groove to concentrate the energy during ultrasonic bonding. The flow of molten polymer is directed towards the groove, which prevents the clogging of the microchannel [109]. Luo et al. proposed a thermal- and solvent-assisted ultrasonic bonding process, which can be performed without an energy director [110]. 

Different studies have been conducted where vibration propagation feedback is obtained from an ultrasonic welder to make the interfacial fusion process more precise. Sun et al. proposed a precise ultrasonic bonding process, in which characteristic waveforms from wavelet packet decomposition were used to analyze the vibration propagation through the thermoplastic polymer. The ultrasonic bonding test bench, which was designed and regulated by recognizing two characteristic points obtained from the characteristic waveform, is as shown in Figure 5C [101]. The fusion bonding interfaces completed at characteristic points I and II are shown in Figure 5D. The proportions of the fusion area to the whole area are about 56% and 63% for characteristic point I, whereas almost the complete interface is fused with the fusion proportions of 97.2% and 96.5% for characteristic point II, as shown in Figure 5D [101]. Similarly, the quality of the ultrasonic weld can also be analyzed through the power and displacement data obtained from the ultrasonic welder. The obtained data could be used to define the best parameters for a specific material and its ultrasonic welding setup [111]. Sun et al. developed a precise control method of ultrasonic bonding, in which the mechanical properties of thermoplastic were obtained by monitoring the propagation of ultrasound while passing through the PMMA substrates [112]. A study was also conducted to observe the vibration propagation during the ultrasonic bonding process of PMMA substrates by theoretical and experimental methods. The variation tendency of vibration propagation was found to be steady, except for the peak-to-peak value of the dynamic force. The relation of ultrasonic propagation to the state of the thermoplastic was also observed [113].

Laser welding is another localized welding method for bonding thermoplastic substrates. Laser Transmission Welding (LTW) involves localized heating at the interface of two thermoplastic substrates to be bonded. Therefore, one of the plastics needs to be optically transparent to the laser radiation, whereas the other must be absorbent. The laser energy that is absorbed in this material causes vibration of the electron bonds, followed by heat transfer to the surroundings through convection and radiation. When heated to temperatures above the T_g_ reaching melting temperature, a weld is formed while applying pressure to increase mating contact forces [114]. Laser welding requires a suitable combination of dissimilar materials with transparent and opaque properties; therefore, it is not suitable for the fabrication of fully transparent microfluidic devices [115]. For generating transparent microfluidic devices, Jiang et al. presented a laser microwelding technique for the assembly of transparent PMMA substrates using an intermediate titanium thin-film spot pattern and a high-power diode laser system with a broad top-hat beam profile. The bonding line is defined by a predetermined metal film spot based pattern as a localized absorber, thus allowing easy control of laser beam alignment in the bonding process [115]. The polycarbonate–polyurethane bonding is obtained by using a continuous-wave fiber laser working at a wavelength of 1064 nm by incorporating carbon black particles in the elastic membrane made of TPE, as well as in one of the sealing foils, to achieve a high optical absorbance [116]. Another method to mitigate the requirement for an absorbing medium to the otherwise mostly transparent thermoplastics is by employing different wavelength ranges, for which the absorption characteristics of the thermoplastics are different, and the use of an absorber is not required. This approach to welding transparent materials uses fs-laser pulses with relatively low pulse energies at much higher repetition rates towards the MHz regime. Heat accumulation is a mechanism generating localized melting in the focal volume. Volpe et al. developed a laser-bonding method to bond two transparent PMMA substrates using a 1030 nm femtosecond fiber laser at a high repetition rate of 5 MHz [117]. Roth et al. demonstrated bonding of COC substrates using an ultrashort pulse laser with a fundamental wavelength of 1028 nm with an adjustable pulse duration from 220 fs to 15 ps and a variable pulse repetition rate up to 610 kHz. In addition, the bonding of PC was also obtained using a laser power of up to 1700 mW, and a mean welding seam width of up to 160 µm was achieved at a welding speed of 40 mm/s [118]. 

The bonding parameters, tools, and bonding results for ultrasonic and laser welding are illustrated in Table 4. Ultrasonic welding is performed by ultrasonic welder with ultrasonic frequencies ranging from 20 to 40 kHz with the power range 300–2000 W. The bonding quality is improved by assistance of a solvent [102,110], or by using tools, such as a self-balancing jig [100,107]. The bonding strength of over 2.5 MPa [15] was obtained using ultrasonic bonding. Laser welding utilizes laser-welding systems to bond two substrates. Laser bonding requires optically transparent substrates along with an absorbent. Thermoplastics are usually transparent and require the assistance of an absorbent material such as titanium film [115] and carbon black particles [116]. Laser bonding can also be achieved without absorbent material through the mechanism of heat accumulation. Transparent thermoplastics are bonded using fs-laser pulses with relatively low pulse energies and higher repetition rates, i.e., 5 MHz [117], 610 kHz [118]. Bonding between two substrates with a maximum tensile strength of 6 MPa [115] is obtained using laser welding.

## 3. Indirect Bonding

### 3.1. Adhesive Bonding

**Figure 6:**
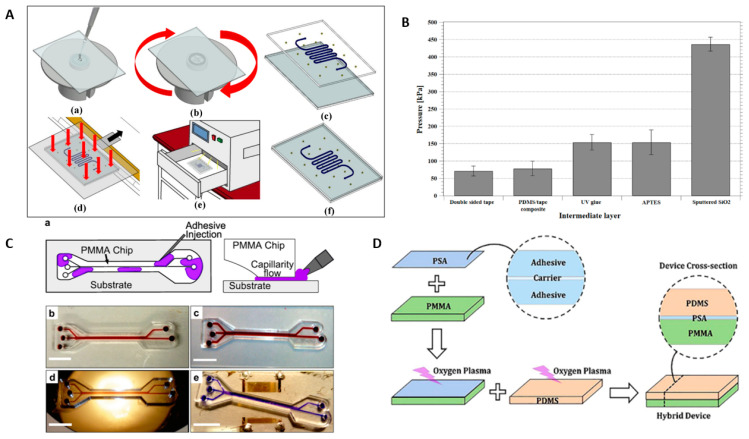
(**A**) Schematic of the adhesive bonding process Reprinted with permission from ref. [123]. Copyright 2016 IOP Publishing Ltd. (**B**) A comparison of the burst pressure for five different intermediate layers (adhesive tape, PDMS/tape, UV glue, APTES, sputtered SiO_2_) Reprinted with permission from ref. [124]. Copyright 2019 IOP Publishing Ltd. (**C**) (**a**) Schematic of the capillary-assisted adhesive delivery method (**b**–**e**) PMMA microfluidic devices bonded via capillarity-assisted adhesive delivery on PMMA (**b**) glass (**c**) silicon (**d**) and LiNbO_3_ (**e**) substrates [121]. (**D**) Schematic of the method for bonding PMMA and PDMS layers at room temperature using pressure-sensitive adhesive (PSA) [94].

In conventional plastic processing, use of adhesive glues to assemble plastic parts is a widely practiced process. This approach is rapid, simple, and cost efficient, with formation of uniform bonds for microfluidic device fabrication. Both liquid form adhesive and dry adhesive are used for thermoplastic bonding. The liquid form adhesive contains synthetic resins with photo or thermal initiators, which when exposed to UV light irradiation or thermal heating, form bonds at the thermoplastic interface. The simplest form of adhesive bonding approach is the direct application of adhesive on the thermoplastic sheet. Recently, different methods, such as spin coating [119], direct adhesive printing [120], capillarity-driven adhesive delivery [121] and selective stamp bonding [122] have been introduced for the application of adhesive on bonding surfaces. One of the adhesive application processes, spin coating, is illustrated in Figure 6A. The UV adhesive was spun on a thermoplastic substrate with micropillars. Then the vacuum-bag method was used for the application of constant pressure for bonding prior to curing by UV exposure [123].

Chip design, surface planarity, surface wettability and adhesive-layer thickness have been noted as primary parameters for producing successful adhesive bonding [125]. The properties of different adhesives were characterized based on different parameters [124,126,127]. The four biocompatible pressure-sensitive adhesives (ARcare 92712, ARcare 90445, ARseal 90880, and ARcare 90106) were compared based on biocompatibility, bond strength, gas permeability, etc. The ARcare 90106 adhesive showed the best result in gas permeability (*p* < 0.001), whereas the ARcare 90445 adhesive displayed the best bond performance [126]. The five different interlayer materials (double-sided tape, a PDMS/tape composite, UV glue, (3-Aminopropyl) triethoxysilane (APTES), and sputter-coated SiO_2_) for bonding PDMS with 3D printed thermoplastics were observed, and the comparison of burst pressure is demonstrated in Figure 6B. The intermediate layer of SiO_2_ demonstrated a good performance in the burst test (i.e., highest burst pressure > 436.65 kPa) compared to other interlayer materials [124]. Hot-melt adhesive produced better bonding than epoxy adhesive while bonding expanded polytetrafluoroethylene (ePTFE) membranes with silicon- and metal-based microfluidic devices [127]. Besides these adhesives, other materials are also used for the bonding of thermoplastics. Wax is used for adhesives in adhesive bonding. Gong et al. reported a wax bonding method that uses paper to absorb the melted wax as an adhesive layer. Excessive wax in the microchannel is purged out with gas pumping. This wax bonding is a reversible bonding process that can be restored upon re-melting the wax [128]. Trinh et al. reported the reversible bonding of PMMA substrates modified by UV irradiation using a biocompatible chitosan (CS)-polydopamine (pDA) hydrogel [129]. A proper bond between a microfluidic gasket and a protein surface was obtained using dual-cured thiol-ene-epoxy polymer without affecting the biological activity [130]. A new bonding technique using 2.5% (*w/w*) PMMA solution as an adhesive layer was developed to seal PMMA microfluidic channels with PC [131]. Fan et al. proposed the bonding of biaxially oriented polystyrene (BOPS)-based microfluidics device using a layer of BOPS or a layer of polyester adhesive film [132]. 

One of the challenges in adhesive bonding is excessive liquid adhesive re-flow into the microchannel, resulting in channel clogging similar to the solvent bonding approach. Several methods have been proposed to prevent adhesive clogging. For example, Salvo et al. spin-coated SU-8 adhesive on a sacrificial carrier, then applied transfer adhesive to the thermoplastic substrate for subsequent adhesive bonding [119]. Dang et al. demonstrated direct adhesive printing to apply adhesive to the PMMA surface and sacrificial microchannels to prevent microchannel clogging. The excessive adhesives and air bubbles were directed into the sacrificial microchannel, preventing microchannel clogging issues [120]. Ku et al. presented a simple bonding method, which prevented channel blockage and microstructure distortion using a pre-patterned self-adhesive film that shields the area of the rigid substrate containing microchannels. Then, an adhesive-assisted sandwich bonding was used to strengthen the bond [133]. A hydrophobic coating was applied on the walls of microchannels before bonding to the prevent clogging of microchannels. The uniform thickness of UV adhesive was maintained using small pillars in one PMMA substrate and spin-coating adhesive on another PMMA substrate [123]. 

The adhesive application method also plays an important role in preventing the clogging of microchannels. Different adhesive delivery methods are practiced to prevent flooding of microchannels. In capillary-assisted adhesive bonding, as illustrated in Figure 6C, adhesive mixture is inserted into the interstitial space between the chip and the substrate, and capillarity forces drive the flow of the adhesive throughout the bonding surfaces without flooding the channels. The interstitial approach utilizes the capillary force to “self-prime” the low-viscosity liquid adhesives into the <10 μm gap, while the thermoplastic cover and microchannel plate are aligned and in close contact. The liquid adhesive automatically wets the thermoplastic interface and “stops” as adhesive reaches the microchannel or microchip edge, due to the capillary pressure drop. Liquid adhesive can be introduced from the microchannel edge or through the hole interfacial method [134], or can be directly delivered into the microchannel, followed by air-purging of the adhesive out of the microchannel [135]. As the liquid adhesive fully fills the gap in the bond interface, it is cured with UV light to complete the bonding process. A capillarity-driven adhesive delivery bonding method was demonstrated using epoxy resin (Araldite Standard) diluted with acetone to achieve bonding between PMMA devices and a variety of substrates, including glass, silicon, and LiNbO_3_ [124]. A 3D printing technique was applied to deposit UV-curable adhesive precisely to bond microfluidic devices and polymer substrates [136]. Wang et al. presented a stamp-and-stick method to prevent the epoxy adhesive from clogging the microfluidic channels while bonding PDMS with PI [136]. Li et al. demonstrated a bonding method that used a soft pressing head to remove the air bubbles from the bonding interface and also to enhance the bonding ratio [137].

Alternatively to liquid adhesive, dry-adhesive films are also used in microfluidic bonding. Dry-adhesive bonding involves bonding of microfluidic devices using pressure-sensitive adhesive, adhesive film, or adhesive tape. Because the adhesive film is “dry”, microchannel clogging issues are minimized. Major process parameters in dry-adhesive bonding include tape thickness, bonding force, and substrate properties. Thicker adhesive tapes are prone to blocking the microchannel. However, with proper force and tape thickness, control tape clogging can be eliminated [138]. More and more commercialized designs have used dry-adhesive film to bond thermoplastic microfluidic devices because this approach is more reliable and straightforward than liquid adhesives. Dry-adhesive films are directly laminated onto the thermoplastic surface to bond the thermoplastic chip [18,139]. The bonding strength of liquid adhesive was reported to be around 10~800 kPa [134,135] for SU-8 adhesive 27 MPa [119], while the bonding strength of dry-adhesive film was reported to be around 32 kPa [139]. However, for dry-film photo-resist, the shear strength of 28 MPa was obtained for 4 min of oxygen plasma treatment while bonding COC substrates [93]. 

One unique advantage of adhesive bonding is its capability for bonding heterogeneous material in “hybrid” microfluidics devices. Bonding PDMS with thermoplastic material enables more microfluidic applications. Figure 6D demonstrates the adhesive bonding method for sealing PMMA and PDMS using double-sided PSA (pressure sensitive adhesive) tape [94]. Tan et al. reported a fabrication of micropumps by bonding PDMS with microchannels with PDMS/PMMA using optically clear adhesive film [140]. Li et al. used a selective stamp-bonding technique to transfer epoxy to bond a PDMS-PS/PET microfluidic device for human lung epithelial cell analysis [122]. A doubly cross-linked nano-adhesive method was reported for sealing PDMS with PI, PET [141]. Adhesive bonding can be practiced to integrate thermoplastic material with paper-based microfluidic devices [142]. Godino demonstrated bonding paper with PMMA using pressure-sensitive adhesive film [143]. Agostini et al. used UV-curable adhesive Norland Optical Adhesive 74 (NOA74) to bond PDMS surfaces functionalized with APTES and untreated plastics and metals [144].

The different adhesives, parameters, tools, and bonding strengths for adhesive bonding are illustrated in Table 5. UV curable adhesive [145], epoxy adhesive [121,122,136], optically clear adhesive [140], PMMA solution [131], etc. are used for bonding thermoplastic substrates. UV curable adhesive needs to be cured by UV irradiation, while epoxy adhesive also needs to be thermally cured at various temperatures. The adhesive is distributed uniformly on the thermoplastic substrates using different methods, such as spin coating [123,131,140,145], capillary-driven method [121], stamp-and-stick method [122,136], etc. Different surface-treatment methods are utilized to improve the bonding strength of adhesive-bonded microfluidic devices. The bonding strength for adhesive bonding varies according to the adhesives used. Chitosan (CS)-Ppolydopamine (pDA) hydrogel adhesive can be used for reversible bonding of PMMA substrates with bonding strength of 0.7 MPa [129]. UV curable adhesive shows the best bonding strength among the adhesives, with maximum bonding strength of 1.35 MPa [145]. For the epoxy adhesive, the maximum bonding strength of 200 ± 92 kPa was obtained [121]. Surface treatment can enhance the bonding strength as observed in COC substrates bonding using ORDYL photoresist in which shear strength of 28 MPa can be achieved, when substrates were treated with oxygen plasma for 4 min. [93].

### 3.2. Chemical Surface Modification 

**Figure 7:**
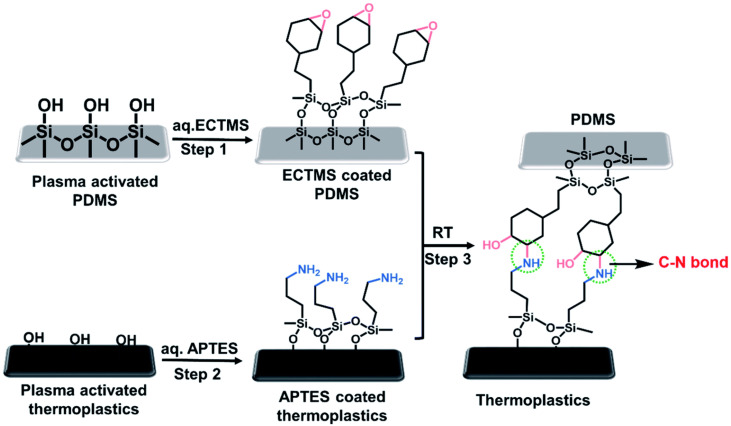
Schematic diagram of bonding of chemically treated PDMS–thermoplastic substrates [147].

Both physical or chemical surface modifications are used for bonding the thermoplastic substrates. While physical surface modification uses UV/ozone or plasma treatment, chemical surface modification bonding uses chemical reagents for activation of thermoplastic surface for bonding. Since there is an additional chemical reagent or layer applied in the bonding interface to increase the surface energy or enhance bonding strength of the microfluidic device, this method is defined as indirect bonding method. For chemical surface modification, chemical reagents such as APTES [147,148], [2-(3,4-epoxycyclohexyl)ethyl]trimethoxysilane (ECTMS) [147], (3-triethoxysilyl)propylsuccinic anhydride (TESPSA) [148] and 3-(trimethoxysilyl)propyl methacrylate (TMSPMA) [149] are used. These chemicals increase surface energy of the substrates and bind them through the formation of strong covalent bond between reagents and substrates. The schematic process for bonding PDMS modified using ECTMS with thermoplastics treated with APTES and ECTMS is shown in Figure 7 [147]. The high quality bond between PDMS and thermoplastics is obtained by the strong covalent carbon–nitrogen bonding using silane reagents. In addition to carbon–nitrogen bonding, the intermolecular hydrogen bond formed between hydroxyl (–OH) and secondary amine (–NH–) groups at the surface interface further increased the bonding strength between PDMS and thermoplastics [147].

Chemical surface modification combined with physical surface treatment can be used to bond similar thermoplastics as well as with PDMS. UV exposure was used for grafting monomer 2-hydroxyethyl methacrylate (HEMA) onto the COC substrates, which increases the bonding strength, surface wettability and compatibility and also bond the substrates in temperature below T_g_ [150]. A thermoplastic sheet containing microchannels was initially activated by corona discharge treatment and later by immersing it in a solution of 6% TMSPMA for 20 min followed by annealing to form bond with PDMS substrates [149]. Plasma activated PDMS can be permanently bonded with amino silane modified thermoplastic surfaces due to the creation of a stable succinimide group in low temperature and pressure [148].

Chemical surface modification can also combine with physical surface treatments for improving the bonding quality of thermoplastic microfluidic devices. O_2_ plasma and ethanol surface treatment helps in fabricating the PET (polyethylene terephthalate) planar nanofluidic at a low bonding temperature of 50 °C [45]. Roy et al. developed a bonding method where the PMMA microfluidic substrates were treated with O_2_ plasma and coated with polyvinyl alcohol (PVA) to obtain thermal bonding at 70 °C without distorting the microchannels [151]. Nguyen et al. assembled the porous PETE membranes after 1 min air plasma treatment modification with 5% GLYMO with PMMA substrates by thermal bonding at 100 °C for 2 min [152]. PMMA substrates were exposed to acetone vapour followed by thermal annealing to improve surface quality and assist in adhesive bonding [121]. PDMS and polyimide substrates were treated with mercaptosilanes and epoxysilanes for the creation of a thiolepoxy bond [153]. 

### 3.3. Microwave Bonding

Microwave bonding technique uses microwave to heat the conductive layer in the bonding interface to achieve bonding between thermoplastic substrates. Thus, it is defined as an indirect bonding method. Due to the localized heating at the bonding interface, which avoids excessive heating of the whole thermoplastic substrate preventing the microchannel deformation. Microwave bonding has less microchannel clogging considerations compare to other bonding method. For conductive layers, several methods have been proposed. Yussuf et al. used a conductive polymer (polyaniline) at bonding interface to achieve the microwave bonding of two PMMA substrates [154]. Lei et al. deposited 100 nm gold/chromium layer on the PMMA substrate and expose it in a 2.4 GHz microwave chamber to locally melting the gold layer for bonding [155]. Toossi et al. report a modified eye-shape gold layer pattern to microwave bond the PMMA chip using commercial microwave oven [156]. Conductive polymer, polyaniline, can be used in the microwave bonding, which does not require additional metal deposition step. Polyaniline can be deposited by screen printing [157] or introduced through interfacial capillary force [158]. Detail thermal analysis of microwave bonding was reported by Dutta et al. that shows the temperature distribution under microwave heating is a function of polyaniline thickness, substrate (PMMA) thickness, and exposure time [159]. 

Typical bonding strength of microwave bonding is 1~2 MPa, and processing time is around 35~120 s. Advantage of the microwave bonding is that it provides selective heating and localized melting through the conductive pattern design and only requires low-cost facility (like household microwave oven) to perform the bonding process. However, this process requires addition metal or conductive layer deposition step that potentially increase the complexity of the fabrication process and microfluidic chip design.

## 4. Conclusions and Future Perspectives

**Figure 8:**
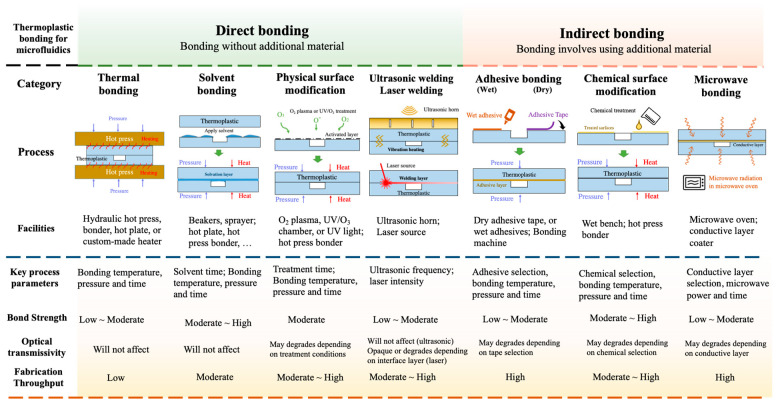
Summarization of direct and indirect bonding methods for thermoplastic microfluidics.

In this review, we provide current thermoplastic bonding technologies for microfluidic devices. Based on the additional materials or layers at the bonding interface, the bonding methods for thermoplastic microfluidic devices can be categorized as direct (no additional materials) or indirect bonding methods (with additional materials). The direct bonding methods including thermal bonding, solvent bonding, physical surface modification and ultrasonic/laser welding and indirect bonding methods including adhesive bonding, chemical surface modification and microwave bonding are surveyed in detail in this review paper. Their bonding mechanism, challenges/solutions, process parameters and required facilities are discussed which are summarized in Figure 8. Surface modification bonding and solvent bonding normally exhibits high bonding strength performance since thermoplastic energies are enhanced by physical/chemical treatments or activated by solvent. For optical performance considerations, since there are no additional materials or layers in the interface, direct bonding such as thermopress, solvent and ultrasonic bonding methods will not affect the bonding performance. However, for other bonding methods like physical/chemical surface modification, laser welding, adhesive bonding or microwave bonding, the bonding may potentially alter the microchannel surface optical properties which may affect the optical performance depending on the extent of surface modification or interface material selection. 

Under extensive development of polymer microfabrication techniques for microfluidics, various thermoplastic bonding methods were developed to meet a broad range of application requirements. Currently, the microfluidic technology has reached maturity, and more and more microfluidic devices are successfully transferred from prototype into commercialized product for real-world applications. Reliable, low-cost, and high throughput thermoplastic bonding methods will be required in this new stage. The microfluidic device will be advanced to a more complex and highly integrated device. The research community will discover more advanced bonding techniques for multilayer, hybrid, and reversible bonding purposes in the coming years. 

## Figures and Tables

**Table 1:** Physical, chemical, and optical properties of commonly used thermoplastics in microfluidics.

Thermoplastic Materials	Physical Properties				Chemical Resistance		Optical Transmissivity		Reference
	Young’s Modulus	T_g_^1^	T_m_^2^	CTE ^3^	Solvent	Acid/Base	Visible	UV	
Polymethylmethacrylate (PMMA)	3200	104–105	130	6–8	good	good	excellent	good	[13]
Polystyrene (PS)	2300–4100	80–90	240–260	10–150	fair	good	excellent	fair	[13]
Cyclic olefin polymers (COC/COP)	2600–3200	65–170	190–320	60–80	excellent	good	excellent	excellent	[14]
Polycarbonate (PC)	2300–2700	145–148	260–270	60–70	good	good	excellent	fair	[15]

^1^ T_g_: glass transition temperature. Unit: °C; ^2^ T_m_: melting temperature. Unit: °C; ^3^ CTE: coefficient of thermal expansion. Unit: 10^−5^ °C^−1.^

**Table 2:** Thermally bonded pairs, parameters, tools required, surface treatment and bonding result.

Bonded Pairs	Parameters			Tools and Experimental Setup	Surface Treatment	Bonding Result	Reference
	T °C	Pressure	Time				
PMMA-PMMA	90	∼5.5 kg/cm^2^	10 min	Spring-driven press	Surface treatment with 5% dibutyl phthalate (DBP) in isopropanol	-	[31]
	∼140	∼6 kg/cm^2^	10 min	Positive temperature coefficient ceramic heater and spring-driven press	Cured with epoxy	-	[32]
	140	2.2 MPa	41 min	Steel plates	82.3 °C isopropyl alcohol for 75 s	185.0 ± 33.3 kPa, and 808.0 ± 80 kPa for untreated and treated samples	[51]
	91–93	1.4–1.9 MPa	360 s	Home-made hot embossing apparatus	Water pretreatment for 1 h	Bonding rate of 96.8%	[49]
	160	1.38 MPa	1 min	GATB using Nanoimprint lithography (NIL) apparatus	Oxygen plasma treatment for 1 min	Failure load 1670 g for GATB at 160 °C and 1.38 MPa	[38]
	95	1–2 MPa	3 min	Interference-assisted bonding with hot embossing equipment	-	-	[50]
	120	Low pressure	1 h	High temperature oven treatment and vacuum dried at 80 °C	-	Microchannels with very low aspect ratios (AR = 1:100)	[39]
PMMA-COC	70	680 kN/m^2^	900 s	Placed in vacuum seal between thermal embosser	Oxygen plasma treatment	Bond strength 67 ± 7 mJ/cm^2^	[46]
PMMA-TPE	70	1.6 MPa	15 min	Pneumatic hot press and electronic pressure regulator	UV surface treatment	Burst load >100 N	[44]
	80	0.52 MPa	-	Hot press machine	Plasma treatment for 1 min	Bonding strength 16 N/cm^2^	[33]
PS-PS	105	0.4 MPa	5 min	Nanostructured plate on PS	-	Deformation ratio 1.1%	[36]
	93.3	6.9 MPa	10 min	Hot press machine	Rinsed with isopropyl alcohol and deionized water	Bonding strength 375.5 kPa	[30]
PI-PI	380–390	100 N	3–5 min	Ceramic heater	-	Bonding strength 80 N	[40]
PET-PET	50	0.15 MPa	15 min	Hot embossing machine	O_2_ Plasma and ethanol treatment	Bonding strength 0.424 MPa	[45]
COC-eCOC	80	2 bar	10 min	Conventional hot press	UV/Ozone treatment for 10 min	Bond strength 445 J/m^2^	[34]

**Table 3:** Solvent bonded pairs, solvent used, parameters, tools required, surface treatment and bonding result.

Bonded Pairs	Solvent Used	Parameters			Tools and Experimental Setup	Surface Treatment	Bonding Result	Reference
		T °C	Pressure	Time				
PMMA-PMMA	Pure isopropyl alcohol	70	No pressure	10 s	Spin coater at 2000 rpm	-	-	[71]
	Chloroform	20	1 atm	12 min	Exposed to CHCl_3_ vapour	O_2_ plasma treatment	Bond strength 38 MPa for double sided exposure	[77]
	Chloroform-Ethanol V_C_:V_E_ = 1:10	40	-	10 min	Soak bonding method	-	Bonding strength 267.5 N/cm^2^	[54]
	Dichloromethane, isopropanol (v:v 2:8)	-	-	10 s	Precision needle-tip applicator	Corona Treatment	Bond strength 2.208 ± 0.001 MPa	[60]
	Chloroform vapour	-	-	10 s	Vapor solvent bonding	UV irradiation	Failure load 3200 N	[74]
	Dichlororethane	-	∼0.2 kg/cm^2^	2 min	Applied by capillary effect using syringe	Cleaned with water and isopropanol	Bond strength 12 MPa	[63]
	Acetic acid	-	-	-	Activated using microwave for 2 min 50 s	-	Bond strength 14.95 ± 0.77 MPa	[65]
	Ethanol (95%)	-	-	56 s	Spin coating at 190 rpm for 10 s	UV irradiation	Bond strength > 10 bar	[69]
	Ethanol	68	120 kPa	15 min	Heated in a fan-assisted oven	Rinsed with isopropyl alcohol and deionized water	Bonding Strength 28.5 MPa	[67]
COP-COP	Cyclohexane	30	3 kN	3 min	Hot Press time of 5 min at 90 °C	-	Microchannel coeficient of variance (CV) 1.4%	[56]
	Dichloromethane	30	1 kN	1 min 30 s	Hot Press time of 5 min at 90 °C	-	CV < 1%	[56]
	Toluene	30	1 kN	4 min 30 s	Hot Press time of 5 min at 90 °C	-	CV < 1%	[56]
PMMA-PS	Acetone with DI water	40	103 kPa	20 min	Pipette and pre-heated hotplate	Rinsed in DI water	Bonding strength 34.4 J/m^2^ for 80% acetone	[66]
PMMA-ABS	Ethanol Solution	-	-	-	Spray coating	UV exposure for 84 s and post annealing at 55 °C	-	[72]

**Table 4:** Ultrasonic and laser welded pairs, parameters, tools required, and bonding result.

Bonded Pairs	Parameters	Tools and Experimental Setup	Bonding Result	Reference
**Ultrasonic Welding**				
PMMA-PMMA	Ultrasonic cleaner Power 300 W, 40 kHz with ultrasound intensity of 0.05 W cm^−3^	Assisted by ethyl alcohol solvent vaporized at 45 °C for 10 min	Bond strength 30.9 mJ cm^−2^ at 60 °C No deformation at 60 °C	[102]
	Frequency 30 kHz speed 50 mm/s Pressure 0.16 MPa time 30 s	Preheating at temperature 75°C	Tensile strength 0.95 MPa	[103]
	frequency of 30 kHz, a power of 1000 W and a maximum amplitude of 60 μm	Ultrasonic welding system (Branson 2000X f/aef),	Burst pressure: 680 kPa	[106]
	Ultrasonic welder 1500 W at 20 kHz, ultrasonic amplitude 60 μm; holding time 5 s Bonding pressure: 24–60 kgf	Self-Balancing jig and energy director	Bonding strength > 2.5 MPa	[107]
	Ultrasonic generator with 20 KHz frequency amplitude 45 μm, 2 layer pressure: 0.25 MPa time 0.6 s 5 layer Pressure 0.45 MPa and time 1 s	Ultrasonic bonding system (Dizo-ultrasonic NC-1800P)	Burst Pressure for two layer linear and serpentine channel and five layer: 553 ± 48 kPa, 572 ± 52 kPa and 417 ± 62 kPa respectively	[109]
	Preheating temperature (°C) 70 Amplitude (μm) 6.6 Trigger pressure (MPa) 0.032 Ultrasonic time (s) 25 Ultrasonic pressure (MPa) 0.276 Holding time (s) 5 Holding pressure (MPa) 0.147	Ultrasonic welding machine (Branson 2000X f/aef, Branson, MI, USA), fixture and hot plate Thermal assisted ultrasonic bonding	tensile strength of 0.95 MPa Dimension loss 0.66% ± 0.60	[110]
	Amplitude (μm) 7.2 Trigger pressure (MPa) 0.033 Ultrasonic time (s) 10 Ultrasonic pressure (MPa) 0.297 Holding time (s) 5 Holding pressure (MPa) 0.297	Ultrasonic welding machine (Branson 2000X f/aef, Branson, MI, USA) Solvent assisted ultrasonic bonding Isopropyl alcohol as solvent	tensile strength 2.25 MPa Dimension loss 0.58% ± 0.55	[110]
	Ultrasonic welder power 2 kW, clamping force 28 kN, Frequency 20 kHz	Ultrasonic welder	No blockage and can withstand 6 bars (gauge) pressure for at least 10 min.	[108]
COP-COP	Ultrasonic welder of Power 750 W frequency 35 kHz	Preheating at 60 °C	--	[97]
	Ultrasonic bonder of frequency 20 kHz, speed 20 mm/s, 90% amplitude for 0.1 s Pressure applied for 10 s	Ultrasonic bonder (Branson, 2000X-aef, USA) Self-balancing jig	No leakage	[100]
**Laser Welding**				
PMMA-PMMA	laser power 25 W beam intensity 70 W/cm^2^ processing time of 15 s output at 970 nm	High power CW diode laser system (LDM 100, Laserlines, Germany) PMMA substrates deposited with titanium film	Tensile strength 6 Mpa	[115]
	Ultrafast fiber laser at a wavelength of 1030 nm and a repetition rate of 5 MHz shortest pulse duration of 650 fs,	Ultrafast fiber laser amplifier	Leakage test upto 1 bar	[117]
PC-TPE	continous wave fiber laser working at a wavelength of 1064 nm	Contour laser welding system (Novolas WS AT from Leister Technologies AG) carbon black particles incorporated in TPE	Average peel strength greater than 0.9 Nmm^−1^	[116]
COC-COC	fundamental wavelength of 1028 nm shortest pulse duration 220 fs pulse repetition rate 610 kHz	Ultrashort pulse laser (Light Conversion, Pharos-10-600)	Leakage test upto 0.6 Mpa for 30 min	[118]

**Table 5:** Adhesive bonding pairs, curing method, coating method, surface treatment and bonding result.

Bonded Pairs	Adhesive Used	Curing Method	Coating Method	Surface Treatment	Bonding Result	Reference
PMMA-PMMA	Chitosan (CS)-Polydopamine (pDA) hydrogel(2:1)	UV irradiation (234 nm, 135 mW cm^−2^)	Using Micropipette	O_2_ plasma treatment	0.7 MPa for 60 s UV exposure and applicable for reversible bonding	[129]
	UV curable (LOCTITE AA 3311) (Acrylated urethane)	UV exposure of 1800 μW/cm^2^ with the peak at 365 nm	Spin coating at 500 rpm thickness around 10 μm	Ultrasonic cleaning	∼1.35 MPa for UV exposure of 30 s	[145]
	Epoxy resin (Araldite Standard)	Cured overnight at room temperature	Capillery driven adhesive	acetone followed by a heat treatment at 70 °C for 15 min	200 ± 92 kPa when cured for 72 h	[121]
	PET film with silicone adhesive and UV curable adhesive	UV curing	Coated into surface	-	364 ± 7 kPa burst pressure	[133]
	UV curable adhesive	UV irradiation for 60 s and vacuum bagging method for uniform pressure	Spin coating at 500 rpm for 10 s followed by 1500 rpm for 20 s	PMMA cleaned with diluted isopropyl alcohol (IPA)	Burst Pressure 10 bar	[123]
	Polyacrylic acid	UV irradiation (234 nm, 135 mW cm^−2^)	Pippette	-	Bond Strength 1.18 Mpa for 60 s UV exposure	[146]
PDMS-PS	PrimeCoat-Epoxy adhesive layer	Cured by heating in oven at 60 °C for 3 h	Selective stamp coating	Oxygen plasma treatment for 30 s	maximum shear stress 2000 Pa	[122]
PMMA-PC	2.5% (*w*/*w*) polymethyl methacrylate (PMMA) solution	dissolved in propylene glycol monomethyl ether acetate (PGMEA)	Spin coated	Annealed in an oven at 80 °C	Bonding strength 0.721 ± 0.03 MPa	[131]
PDMS-PI	Epoxy adhesive	Cured in hotplate at 60 °C for 2 h	Stamp and stick	PDMS treated with oxygen plasma for 30 s	Peeling force 5 N Bonding strength 100 kPa	[136]
PDMS-PMMA	ARclear^®^ Optically clear adhesive 8154	Thermal curing at 80 °C for 1 h followed by oxygen plasma	Spin coating at 1500 rpm for 30 s	Washed with ethanol and deionised water	Bond strength > 20 kPa	[140]
COC-COC	ORDYL photoresist	baked for 2 min at 80 °C on a hotplate	Manually laminated	oxygen plasma treatment for 4 min	shear strength 28 MPa	[93]

## Data Availability

Not applicable.
